# Predictors of professional placement outcome: cultural background, English speaking and international student status

**DOI:** 10.1007/s40037-016-0289-x

**Published:** 2016-08-04

**Authors:** Stacie Attrill, Sue McAllister, Michelle Lincoln

**Affiliations:** 1 The University of Sydney, Sydney, Australia; 2 Flinders University, Adelaide, Australia

**Keywords:** Clinical education, International student, Placement, Acculturation theory

## Abstract

Placements provide opportunities for students to develop practice skills in professional settings. Learning in placements may be challenging for culturally and linguistically diverse (CALD) students, international students, or those without sufficient English proficiency for professional practice. This study investigated whether these factors, which are hypothesized to influence acculturation, predict poor placement outcome. Placement outcome data were collected for 854 students who completed 2747 placements. Placement outcome was categorized into ‘Pass’ or ‘At risk’ categories. Multilevel binomial regression analysis was used to determine whether being CALD, an international student, speaking ‘English as an additional language’, or a ‘Language other than English at home’ predicted placement outcome. In multiple multilevel analysis speaking English as an additional language and being an international student were significant predictors of ‘at risk’ placements, but other variables tested were not. Effect sizes were small indicating untested factors also influenced placement outcome. These results suggest that students’ English as an additional language or international student status influences success in placements. The extent of acculturation may explain the differences in placement outcome for the groups tested. This suggests that learning needs for placement may differ for students undertaking more acculturative adjustments. Further research is needed to understand this and to identify placement support strategies.

## What this paper adds

Student characteristics such as being from a culturally and linguistically diverse background, being an international student, speaking English as an additional language or speaking a language other than English at home are acculturative factors that may predict poor academic performance. However, their influence on students’ performance in professional placements has not been investigated. The results of this study suggest that being an international student or speaking English as an additional language predicted an ‘at risk’ placement outcome, but other acculturative factors tested did not. Acculturation theory is a useful lens to examine the differences between these groups of students.

## Introduction

Research conducted in Western contexts has found that students from culturally diverse and non-English-speaking backgrounds perform poorly in university degrees for health professionals compared with students from the dominant culture who speak English [1]. Such students may be domestic students from culturally and linguistically diverse (CALD) backgrounds or international students who are not residents of their country of study. In 2014, there were 4.5 million international students globally, and 53 % of these were from Asia [2, 3]. In Australia, where this study was conducted, international students comprise 20 % of university students, but limited data exist about the participation of domestic CALD students in higher education [3]. Studies of health professional students have established that being CALD [1] and speaking English as an additional language [4, 5] are predictors of poor academic performance, but the relationship for international students is unclear. Whilst previous studies have focussed on academic outcomes, little is known about the performance of these students in their professional placements.

Health professional education programmes require students to undertake placements situated within a range of health and community settings. These provide opportunities to work directly, and in an authentic way, with clients and situations related to the placement setting [6]. Placements facilitate opportunities for students to develop and practice professional knowledge, attitudes and skills, embedded within the sociocultural practices of their profession [7]. In many health professions, students are supervised in their placements by qualified practitioners who provide direct educational support and workplace based competency assessment during the placement [8, 9]. Findings from qualitative studies suggest that in Western, English-speaking contexts CALD students, international students or those speaking English as an additional language may experience learning challenges in placements [10–13].

To succeed in these placements, international students [10, 14] and domestic CALD students [13, 15] must adjust to Western settings; manage culturally unfamiliar approaches to learning, assessment and supervision; and conduct their practice in English. Acculturation describes the process of cultural and psychological change that occurs following contact between individuals or groups from dissimilar cultures. This process of adjustment may influence learning outcomes for students in placements [16]. The extent of an individual’s acculturation is influenced by several factors including their ethnic and cultural similarity to the receiving community, proficiency in the majority language, social supports, adaptation strategies, and the attitudes of the host culture [16–18]. Students who must undertake multiple or extensive adjustments may experience acculturative stress that affects their ability to cope with these changes [17] and impacts their academic performance [18]. In placements, all students must adjust for the context and practice of each setting. However, students who are also undertaking extensive acculturative adjustments may need to complete additional learning [10, 18] and this may influence their placement learning outcomes.

Previous studies have used acculturation to explain educational, social, cultural and language adjustments made by international students, and how stress related to acculturation affects adaptation to learning at university, but have not investigated the critical learning environment in placements [18]. Acculturation may also affect domestic CALD students as these changes are known to continue for several generations following migration [17]. This suggests that acculturative factors may also impact educational outcomes for these students [18]. Studies identifying that international and domestic students who are CALD experience difficulties learning on placement and meeting performance expectations are small and preliminary in nature [11, 13, 15]. Students’ placement success does relate to their English as this can impact communication with patients and health teams [12, 14]. Speaking English as an additional language is an acculturative factor that has been identified to predict academic performance for health professional students [4, 5, 18]. However, speaking a language other than English at home (LOTEH) includes native English speakers, and is also a contextual variable that affects acculturation [17], but is rarely included in educational studies. Speaking English as an additional language or a LOTEH may be permutations of English language status that predict placement outcome. Factors related to students’ cultural background or international student status may also hinder placement performance due to the process of acculturation, but little empirical data exist to support this [11, 15].

Few studies have examined the predictive nature of CALD, being an international student, speaking English as an additional language and speaking a LOTEH together, and no previous research has investigated these for professional placement outcomes [1, 4, 19]. Investigating outcomes in placements is critical as they are key for the preparation of health professionals who can function competently in the workplace [6]. The placement context may pose additional acculturative challenges for students who are culturally and linguistically diverse [14]. This study aims to determine whether factors known to influence acculturation [17], including students’ background or English status, predict their outcomes in professional placements.

We hypothesized the following:

- English status categories (‘English as an additional language’ and ‘LOTEH’) and background status categories (‘international students’ and ‘CALD’) are separately predictive of poor placement outcome compared with English as first language, domestic or non-CALD students.
- Interactions between background and English status variables will additionally predict ‘at risk’ placement outcome.

## Method

### Context

This prospective cohort study was conducted with speech-language pathology students in Australia, an English dominant Western culture.

#### Participants

Students were recruited from five undergraduate and masters professional preparation speech-language pathology programmes at three universities in different cities. There were 854 students who consented, a consent rate of 82.2 %.

#### Data collection

Placement outcome data for any completed placements were collected from a staff member coordinating placement courses (placement coordinator) at each university. Data from six placements were excluded due to incompleteness; data from 2747 placements from 852 students were analyzed.

Each university utilized the same standardized and validated assessment of speech-language pathology student competency development, which is conducted in the placement by the student’s supervisor [20, 21]. Continuous formative assessment of competency development occurs over the placement and a final summative assessment is recorded [9]. Placement outcome is determined by measuring competency development yielded by this assessment tool and qualitative appraisal by the supervisor. If the quantitative and qualitative judgments are not aligned with the required level of performance to pass, the placement outcome is determined via university mediation which also considers relevant contextual variables.

Placement outcome data were collected by the placement coordinator and one member of the research team and coded into one of four categories:

- Pass: Student passed the placement.
- Additional support: Student received additional learning support, such as mentoring or learning contracts during their placement due to competency development concerns, and subsequently passed the placement.
- Supplementary placement: Student required an extension of placement time or a supplementary placement due to competency development concerns, and subsequently passed the placement.
- Fail: Student failed the placement and was subsequently required to repeat.

The ‘Pass’ category comprised 2534 placements (92.2 %). Students in the remaining three categories had each been identified by their placement supervisor as being at risk of not meeting placement competency requirements and had received additional university support. These ‘at risk’ categories were small: ‘Fail’ had 71 placements (2.6 %), ‘Supplementary placement’ had 17 placements (0.6 %) and ‘Additional support’ had 125 placements (4.6 %). Initial modelling using these categories was not meaningful due to estimation problems and high standard error. Therefore, they were combined into a single ‘at risk’ category. This combined ‘at risk’ category defined poor placement outcome, and comprised 213 placements (7.8 %). The alternative placement outcome category was ‘Pass’.

Students complete several placements during their speech-language pathology programme, and these occur across two or three years, depending on the university. This study captured placements over three consecutive years from 2011–2013, but during this period most students contributed data over one year (44.4 %) or two years (45.8 %). The mean number of placements for all students was 3.22 (SD = 1.6; range = 1–7). Unpaired T‑tests revealed no significant differences between the mean number of placements for all students compared with the mean placements for each predictor. Each student was assigned a university identifier and a programme variable for undergraduate or masters enrolment.

#### Student background survey

A survey was constructed to collect students’ cultural and linguistic characteristics using relevant standard questions recommended by the Australian Bureau of Statistics [22]. Acculturative factors were classified into five predictors shown in Table 1, and coded into binary ‘yes/no’ responses. These were defined as:

1. English language status:
  English as an additional language: students for whom English is not their first language.
  LOTEH: students who use a language other than English at home.
2. Background status:
  ‘Defined CALD’: students born outside of Australia who speak LOTEH [22];
  ‘Perceived CALD’: Students identifying as CALD, including those Australian-born students perceiving as belonging to the cultural group/s of their family.
  International students: students who are not Australian residents [2].

**Table 1:** Survey question derived and proportion of students per predictor

Survey question	Predictor	Number of students identified (% cohort)
1. Do you consider yourself to come from a culturally and linguistically diverse (CALD) background?	Perceived CALD	270 (31.7)
2. In which country were you born?	Defined CALD^a^	133 (15.6)
2b. Are you an international student enrolled in an Australian University programme?	International student	106 (12.4)
5. Which language did you *first* speak as a child?	English as an additional language	127 (14.9)
6. Do you speak a language other than English at home?	Language other than English at home (LOTEH)	219 (25.7)

^a^Defined CALD identified with data from survey questions 2 and 6.

Students present in combinations of these groups, depending on their individual background and English status. For example, an international student may be a native English speaker, but have a CALD background.

#### Analysis

All data analyses were conducted in SPSS v22. Frequency data for each predictor were gathered to identify the proportion of placements with a ‘Pass’ or ‘At risk’ outcome, as shown in Table 2. Students’ country of origin was also collated.

**Table 2:** Placement outcome data according to predictor variables

	Predictor	Category	Placement outcome *N *(%)		Total placements *N *(%)
			Pass	At risk	
Student predictors	Perceived CALD Status	Perceived CALD	794 (87.9)	109 (12.1)	903 (32.8)
		Not perceived CALD	1740 (94.4)	104 (5.6)	1844 (67.1)
	Defined CALD	CALD	370 (86.3)	59 (13.8)	429 (15.6)
		Not CALD	2164 (93.4)	154 (6.6)	2318 (84.4)
	IS^a^	International student	283 (85.2)	49 (14.8)	332 (12.1)
		Domestic student	2251 (93.2)	164 (6.8)	2415 (87.9)
	EAL^b^	EAL	336 (83.8)	65 (16.2)	401 (14.6)
		EFL^d^	2198 (93.7)	148 (6.3)	2346 (85.4)
	LOTEH^c^	LOTEH	655 (88.2)	88 (11.8)	743 (27.0)
		English only	1879 (93.8)	125 (6.2)	2004 (73.0)
Interactions between predictors	IS^a^ and EAL^b^		92 (76)	29 (24)	121 (4.4)
	Perceived CALD and EAL^b^		307 (82.5)	65 (17.5)	372 (13.5)
	Perceived CALD and LOTEH^c^		576 (87.0)	86 (13.0)	662 (24.1)
	IS^a^ and LOTEH^c^		243 (85.3)	42 (14.7)	285 (10.4)
Control predictors	University ID	A	563 (91.5)	52 (8.5)	615 (22.4)
		B	1294 (91.3)	123 (8.7)	1417 (51.6)
		C	677 (94.7)	38 (5.3)	715 (26.0)
	Programme	Undergraduate	1818 (93.1)	134 (6.9)	1952 (71.1)
		Masters	716 (90.1)	79 (9.9)	795 (28.9)
	Year (of placement assessment)	2011	701 (93.3)	50 (6.7)	751 (100)
		2012	794 (89.6)	92 (10.4)	886 (100)
		2013	1039 (93.6)	71 (6.4)	1110 (100)
*Total placements*			*2534 (92.2)*	*213 (7.8)*	*2747 (100)*

*CALD* culturally and linguistically diverse

^a^International students

^b^English as an additional language

^c^Language other than English at home

^d^English as a first language

Binomial logistic regression analysis was used to estimate the odds ratio for each predictor in relation to an ‘At risk’ placement outcome [23]. Multilevel modelling was applied using the Generalized Mixed Linear Models procedure as each student contributed data from multiple placements during the study, creating a nesting effect. This ensured findings were not artificially inflated by individual students’ performance [23, 24]. The predictors were tested for multicollinearity using polychoric correlation and variance inflation factor prior to analysis [25]. The ‘defined CALD’ variable had a collinear relationship with ‘perceived CALD’ (>0.9) and ‘English as an additional language’ variables, and was removed from analysis [23, 26].

Table 3 shows the models of single and multiple predictors of ‘At risk’ placement outcome. University, programme and placement year variables were controlled in each iteration. The simple multilevel logistic model investigated the likelihood that the individual variables predicted ‘at risk’ placement. These predictors were then entered together using multiple logistic multilevel analysis in model 1. In model 2, interactions between ‘international student’, ‘perceived CALD’, ‘English as an additional language’ and ‘LOTEH’ variables suggested to impact placement performance [11, 13] were entered with the single predictors from model 1. Non-significant student predictors were removed one-by-one via backwards elimination for the final model [26].

**Table 3:** Odds ratios for variables predicting ‘at risk’ placement

		Predictor OR of ‘at risk’ placement outcome (CI = 95 %)			
	Predictor	Predictors entered individually	Model 1: Single predictors entered together	Model 2: Single predictors entered with interactions	Final model: Non-significant student predictors removed
Student predictors	English as an additional language (EAL)	2.75 (1.82–4.17)***	2.19 (1.27–3.76)**	1.75 (0.90–3.53)	2.38 (1.55–3.67)***
	Language other than English at home (LOTEH)	1.99 (1.38–2.89)***	0.80 (0.44–1.46)	0.83 (0.45–1.54)	–
	International student (IS)	2.33 (1.48–3.66)***	1.71 (1.00–2.91)*	1.34 (0.65–2.77)	1.83 (1.14–2.92)*
	Perceived CALD	2.19 (1.53–3.14)***	1.48 (0.86–2.56)	1.57 (0.90–2.75)	–
Interactions between predictors	IS and EAL	–	–	1.66 (0.59–4.65)	–
	IS and LOTEH^a^	–	–	Estimation problem/high standard error	–
	Perceived CALD and EAL^a^	–	–		–
	Perceived CALD and LOTEH^a^	–	–		–

Unconditional (Null) OR = 0.07 (0.06–0.09)***, variance estimate = 1.31, median odds ratio = 2.98

Final model OR = 0.04 (0.02–0.07)***, variance estimate = 1.21, MOR = 2.86

Year, University and Programme variables were entered as controls for individual predictors and for each model iteration

*OR* odds ratio, *CALD* culturally and linguistically diverse

^a^Interactions between IS and LOTEH; Perceived CALD and EAL; Perceived CALD and LOTEH were eliminated from Model 2 due to estimation problems and standard error

*p ≤ 0.05, **p ≤ 0.01, ***p ≤ 0.001, *N* = 2747, common subjects = 852

## Results

The proportion of students for each predictor group are displayed in Table 1. The majority of international students were from countries in South-Eastern Asia (Singapore = 43.4 %; Hong Kong (SAR of China) = 15.1 %; Malaysia = 10.4 %); whereas ‘perceived CALD’ students were predominantly from Australia (43.0 %), followed by Singapore (16.7 %) and Hong Kong (10.4 %).

‘Perceived CALD’, ‘defined CALD’, ‘international student’, ‘English as an additional language’ and ‘LOTEH’ predictors had proportionately more ‘at risk’ placement outcomes than their alternative categories, as demonstrated in Table 2. Interactions between background and English status predictors had proportionately more ‘at risk’ placements than those from single predictors.

The unconditional model revealed that the odds of any student having an ‘at risk’ placement outcome was small but significant (OR = 0.07;* p* ≤ 0.001), as shown in Table 3. All predictors tested in the simple multilevel model were significant. However, ‘international student’ and ‘English as an additional language’ were the only significant student predictors of ‘at risk’ placement outcome in the final multiple predictor model. In this model, international students were 1.83 times more likely to have an ‘at risk’ placement than domestic students (*p* < 0.05); and English as an additional language students were 2.38 times more likely than English as a first language students (*p* < 0.001). Interactions between background and English status predictors were tested in the multiple multilevel analysis, but ‘perceived CALD and English as an additional language’, ‘Perceived CALD and LOTEH’ and ‘International student and LOTEH’ interactions were eliminated due to estimation problems and high standard error. The remaining interaction between ‘international student and English as an additional language’ was not significant and did not enhance the model. University and programme variables used as controls were not significant, except for ‘Year of placement’ where students who completed a placement in 2012 were significantly more likely to have an ‘at risk’ placement in each model iteration, and 1.63 times in the final model (*p* < 0.01).

Atkinson’s *R* was calculated to determine the importance of the ‘international student’ and ‘English as an additional language’ predictors to the placement outcome model. Although the ‘international student’ and ‘English as an additional language’ variables were significant predictors of ‘at risk’ placement, their *R* values of 0.02 and 0.03 respectively indicate that these cultural and linguistic characteristics have a small contribution to placement outcome overall [24]. The *R* value for 2012 placement year was 0.02.

The median odds ratio was calculated to determine the extent that the individual probability of an ‘at risk’ placement outcome was influenced by heterogeneity among students [27, 28]. The median odds ratio value of 2.86 in the final model indicated that the residual heterogeneity among students was of greater relevance for placement outcome than ‘international student’ or ‘English as an additional language’ status.

CALD and international student groups are rarely delineated in previous studies, and differences between these variables in the multiple multilevel analysis prompted investigation of the proportion of ‘perceived CALD’ and international students who also spoke English as an additional language. Post-hoc analysis conducted using the ‘*N*-1’chi-squared test found no significant difference in the proportion of ‘English as an additional language’ students in the ‘international student’ and ‘perceived CALD’ groups (χ^2^ = 0.150; *p* = 0.7) [29].

## Discussion

In this study, acculturation theory was used as a lens to identify predictors of placement outcome for health professional students studying in a Western, English-speaking country. Specifically, background status variables of ‘perceived CALD’ or ‘international student’ and English status variables of ‘English as an additional language’ or ‘LOTEH’ were modelled to determine if they predicted poor placement outcome. We also investigated whether interactions between background and English status variables additionally predicted ‘at risk’ placement outcome. Interactions between ‘international student and English as an additional language’ were not significant in the multiple predictor model, but estimation of other interactions was limited.

In the simple multilevel logistic analysis, the single variables each significantly predicted ‘at risk’ placements. However, only ‘English as an additional language’, ‘international student’ and ‘placements in 2012’ remained significant predictors in the final model which utilized a more powerful multiple multilevel logistic analysis [23, 26]. Longitudinal data are required to determine if the variation in ‘at risk’ placement outcome in 2012 is more than anomalous. Whilst these variables were significant predictors of ‘at risk’ placement, their importance for the overall placement outcome was small, and the median odds ratio indicated that heterogeneity among individual students is also a relevant factor for placement outcome [27]. Placement outcome is likely to be predicted by many untested factors related to student academic performance, placement setting, supervision and assessment. This study did not intend to model a broad range of variables, but rather to determine whether the acculturative factors identified predicted placement outcome. The contribution of the ‘international student’ and ‘English as an additional language’ predictors in the multiple multilevel analysis indicates that these should be included in future studies. Individual acculturative factors such as personal attributes and values, sociodemographic status and coping strategies may also contribute to placement outcome [17, 18], and should also be considered for future studies.

Students who speak English as an additional language were more likely to have an ‘at risk’ placement outcome but students speaking a LOTEH were not. This finding extends previous research linking speaking English as an additional language with poor academic performance [4, 5] into the professional placement context and lends support to qualitative findings identifying English proficiency as influencing students’ placement success [10, 13, 14]. Acculturation theory predicts that challenges associated with using English as an additional language result in stressors for students which inhibit their adjustment to new educational environments, even when their cultural background is constant [17]. Adjustments for language are likely to be greater for students who speak English as an additional language than students who speak a LOTEH, as this includes native English speakers. Adjustments may be intensified in placement settings, where the client focussed nature of services reduce opportunities to scaffold students’ professional communication skills [6]. Whilst speaking English as an additional language has been linked with poor academic performance [4, 5], it is not a direct measure of English proficiency. Measures of proficiency may better reflect communication challenges in placement settings as speaking English as an additional language does not reflect a students’ application of English [11, 13, 15]. Further research is needed to examine the relationship between English as an additional language, English proficiency and placement outcome.

Previous studies have identified CALD students as having a greater risk of academic difficulties, but these studies have not clearly differentiated between CALD and international students or addressed placement outcomes [1]. In this study, being an international student was more predictive of ‘at risk’ placement outcome than belonging to ‘perceived CALD’. As ‘English as an additional language’ also predicted ‘at risk’ placement outcome, differences in the proportion of English as an additional language speakers in the ‘international student’ and ‘perceived CALD’ groups may have assisted to explain their difference in placement outcome. However, they were not significantly different. These groups were distinguished by domestic students born in Australia, who comprised almost half of the ‘perceived CALD’ group. The identification of these Australian students as CALD may relate to the cultural background of their family. Domestic CALD students were also included in study cohorts of previous research that identified CALD as a factor predicting academic failure [1, 19]. The factors that influence the extent of a student’s acculturation to the receiving culture are likely to impact international students differently to domestic CALD students. These include their prior education experiences, cultural similarity with and fluency in the language of the host country [17]. This may account for the finding that being an international student predicted ‘at risk’ placement performance, but being ‘perceived CALD’ did not. The time limited and specific intentions of the international students’ sojourn [18] or the greater acculturation of domestic CALD students to the home culture [17] may also influence outcomes.

Domestic CALD students have been noted to experience challenges in professional placements [13]. However, international students may need to undertake greater acculturative adjustment to operate successfully within these environments compared with those of their home culture. These factors may be independent of cultural or language background, as students from Western, English-speaking backgrounds also undertake adjustment to accommodate their learning in foreign professional placement settings [10]. International students may therefore find adjustment to the language, learning, cultural and/or organizational expectations of professional placements more challenging than their domestic peers, regardless of their background [10, 11, 14]. This may have a greater contribution to international students’ placement outcomes than previously understood. Acculturative factors may therefore assist to explain differences in placement outcomes between the ‘international student’ and ‘perceived CALD’ groups; however, further research is needed to confirm this.

Frequency findings for interactions between ‘international student and English as an additional language’ predictors indicated that these students may be more likely to have an ‘at risk’ placement outcome than other student groups. International students who also speak English as an additional language may need to undertake more extensive acculturative adjustments than those represented in the other groups [16, 18]. However, in the multiple multilevel analysis, interactions between ‘international student and English as an additional language’ were not significant. Therefore, students who are international students and speak English as an additional language are not more likely to have an ‘at risk’ placement outcome than students who are either international students or speak English as an additional language. This suggests that the ‘international student’ and ‘English as an additional language’ variables independently predict placement outcome, and both should be included in future studies of placement outcome.

These findings that suggest that international students and students who speak English as an additional language may require support for their learning and performance in placements. However, there is little empirical evidence of what strategies would be effective. Future research should clarify learning needs for these students and explore appropriate placement supports [10, 13].

### Limitations

The identity, background and placement outcome of students who did not consent for this study is unknown, so it is not possible to determine if the results represent speech-language pathology students from the participating universities. However, consent rates did not markedly differ across the three universities, and the proportion of international and domestic students within this study approximates that identified in previous speech-language pathology research [30].

There are likely to be many predictors of placement outcome including factors related to student, supervisor, placement context and the assessment of competency, but this study did not aim to include these in modelling. Future studies should include a broader range of predictors, including ‘international student’ and ‘English as an additional language’ to determine a model of placement outcome.

Backwards elimination was utilized to remove non-significant predictors from the modelling. While this potentially introduces bias, it is an accepted strategy where research does not aim to identify a causal model [26]. Multicollinearity limited modelling of students in the ‘defined CALD’ group, which constrained the analysis of CALD as a predictor. Estimation problems and standard error also precluded investigation of interactions between predictors in the multiple multilevel analysis.

## Conclusion

This study aimed to identify if students’ placement outcomes are predicted by being an international student, CALD or English-speaking status. ‘English as an additional language’ and ‘international student’ variables were significant predictors of ‘at risk’ placement outcome, but their relative contribution to overall placement outcome was small. Acculturation theory may be useful to advance understanding of factors that influence students’ success in their professional placement programmes. Future research should clarify the impact of speaking English as an additional language or being an international student on placement outcomes, and strategies that contribute to placement success for these students.
